# Office-Based Carpal Tunnel Release Using Ultrasound Guidance in a Community Setting: Long-Term Results

**DOI:** 10.7759/cureus.27169

**Published:** 2022-07-23

**Authors:** Russell A Bergum, Mark R Ciota

**Affiliations:** 1 Orthopedics, Interventional Orthopedic Solutions, Rochester, USA; 2 Orthopedic Surgery, Mayo Clinic, Albert Lea, USA

**Keywords:** simultaneous bilateral, ultrasound-guided, office-based, carpal tunnel syndrome, carpal tunnel release

## Abstract

Objectives: To report the safety and effectiveness of office-based carpal tunnel release (CTR) using ultrasound (US) guidance in a general community population.

Methods: This was a prospective single-center study that evaluated patients treated with CTR using US guidance between March 2019 and August 2020 for whom one-year data were available. Procedures were performed in an office-based procedure room using local anesthesia. Main outcomes of this study included complications, Boston Carpal Tunnel Questionnaire scores (BCTQ-SSS, BCTQ-FSS) and Quick Disabilities of the Shoulder and Hand (QDASH) scores.

Results: Among 88 patients (123 hands) aged 30 to 89 years with available one-year data, 29 patients had simultaneous bilateral procedures. No intraoperative complications were reported. Statistically significant and clinically important improvements in BCTQ-SSS, BCTQ-FSS and QDASH scores occurred within two weeks and persisted through one year of follow-up (p < 0.001). Outcomes were similar for simultaneous bilateral releases. During follow-up, one patient developed complex regional pain syndrome that was successfully treated and subsequently had CTR using US guidance on the contralateral hand. No other complications were observed during one-year follow-up.

Conclusion: Office-based CTR using US guidance is safe, effective and provides sustained clinical improvements at one-year follow-up in a community population. Simultaneous bilateral procedures were well-tolerated and resulted in similar clinical improvements.

## Introduction

Carpal tunnel syndrome (CTS) is a peripheral nerve entrapment syndrome that affects 3% to 6% of adults in the United States resulting in annual healthcare costs of over 2 billion dollars [1-5]. Carpal tunnel release (CTR) is an effective treatment option for patients with severe CTS symptoms with over 90% of patients reporting clinical improvement [3,4,6-8]. Historically, CTR was performed via a 3-5 cm palmar incision. More recently, less invasive CTR techniques have been introduced that reduce postoperative pain, promote quicker recovery, and improve cosmesis [6-10].

Ultrasound (US) is an excellent soft tissue imaging modality that provides the opportunity to perform CTR through small incisions (< 5 mm) while dynamically visualizing the at-risk structures in the carpal tunnel region. Although multiple publications have documented the safety and effectiveness of CTR using US guidance [11-23], no previous study has reported the long-term results of procedures performed in an office-based procedure room within a rural community practice. The objective of this study was to document the one-year clinical outcomes of office-based CTR using US in a general community population.

## Materials and methods

Study design

This was a prospective single-center study that evaluated patients treated with office-based CTR using US guidance for whom one-year follow-up data were available. Study procedures and methodology followed the principles set forth in the Helsinki Declaration. This study was reviewed and approved by the Institutional Review Board affiliated with the authors’ practice (Mayo Clinic IRB#18-010140). Written informed consent was obtained from all patients.

Study participants

The authors' practice in a general rural community practice setting serving approximately 50,000 people. All patients meeting the following criteria were offered the option of receiving office-based CTR using US guidance as part of a shared decision-making process: (1) clinical diagnosis of CTS; (2) a minimum of six months nonsurgical management; and (3) confirmatory electrodiagnostic testing consistent with median neuropathy at the wrist. All eligible patients were subsequently evaluated with US to detect anatomic variations that would preclude the procedure. No patients were excluded based on US findings.

Procedural description

Prior to the study, the first author had 10 years of experience in diagnostic and interventional ultrasound and the second author had one year of experience in interventional ultrasound. Each author completed a formal, cadaver-based training program for CTR-US prior to starting the procedure. All procedures were performed in an office-based procedure room using sterile technique (including sterile US probe cover and sterile gel), local anesthesia without a tourniquet, a high frequency linear array transducer (4-18 MHz or 3-14 MHz) and one of two cart-based US machines (Samsung RS80A or HS60, Samsung HME, Ridgefield Park, NJ, USA).

**Figure 1:**
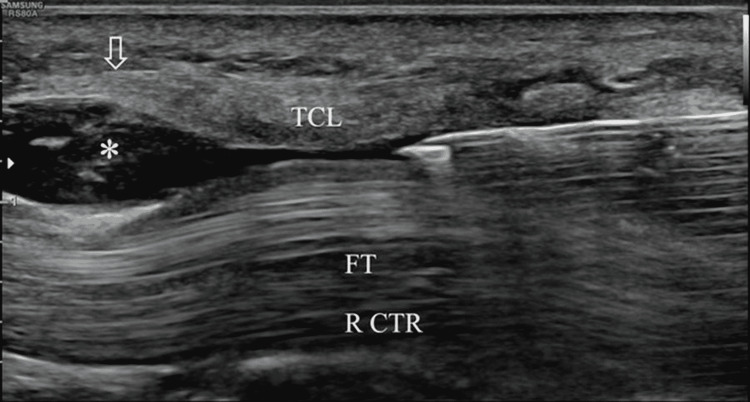
Longitudinal ultrasound view of the carpal tunnel demonstrating hydrodissection of the transverse carpal ligament (TCL) with 1% lidocaine (Left = distal). The needle is positioned just deep to the TCL. Injected fluid (asterisk) separates the underlying flexor tendons (FT) from the TCL. Vertical arrow = location of distal TCL.

The patient was placed supine with the arm abducted and the hand resting comfortably on the arm board or other supportive stand with the wrist slightly extended. The carpal tunnel was scanned to identify relevant anatomic landmarks including the median nerve, thenar motor branch, digital nerves, ulnar artery and superficial arch, transverse carpal ligament (TCL), transverse safe zone (TSZ, distance between the median nerve and either ulnar artery or hook of hamate - whichever is closer), and any anatomic variations that would affect the procedure. Local anesthesia was obtained using direct US guidance and standard 22- or 25-gauge stainless steel needles to deliver 5-15 ml of 1% lidocaine without epinephrine subcutaneously at the incision site and into the carpal tunnel. Local anesthetic was also used to hydrodissect the synovial tissue, median nerve, and flexor tendons away from the TCL and further delineate the boundaries of the ligament (Figure 1). Liberal use of ultrasound-guided hydrodissection of the TCL from the palmar fascia and hydrodissection of the TCL from the flexor tendons allows the cutting blade to more easily transect the TCL without extending into the palmar fascia.

**Figure 2:**
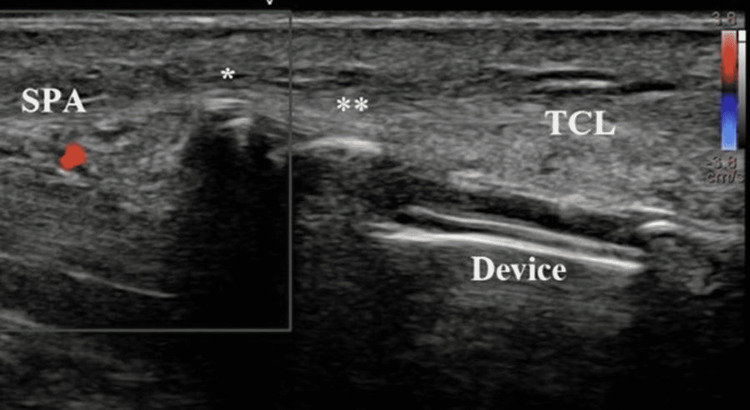
Similar view to Figure 1 with Doppler imaging. The device is positioned directly deep to the transverse carpal ligament (TCL). Both the tip (asterisk) and recessed blade (double asterisk) are easily seen, as is the superficial palmar arterial arch (SPA, appearing as red due to Doppler signal).

A #15 scalpel blade was then used to make an incision < 5 mm length at the proximal wrist crease, passing through the pre-anesthetized skin wheal and antebrachial fascia. Following this, US guidance was used to pass a 3-4 mm blunt-tipped elevator into the carpal tunnel to perform blunt dissection. Thereafter, CTR was performed using a commercially available retrograde knife (SX-One MicroKnife, Sonex Health, Inc., Eagan, MN, USA). US guidance was used to position the device within the TSZ at the distal TCL (Figure 2).

**Figure 3:**
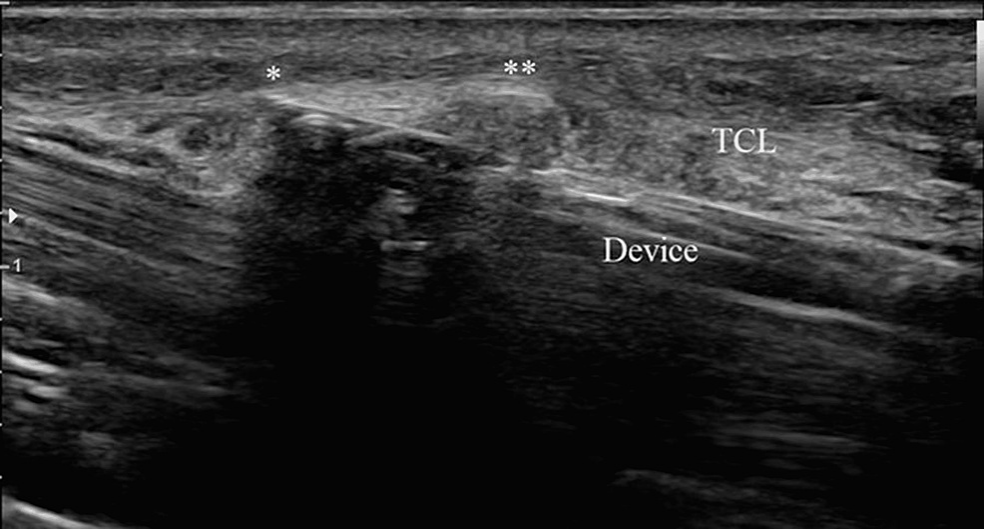
Similar view to Figures 1, 2. The device tip is visible (asterisk), and the blade (double asterisk) has been deployed to cut the transverse carpal ligament (TCL) distal to proximal.

**Figure 4:**
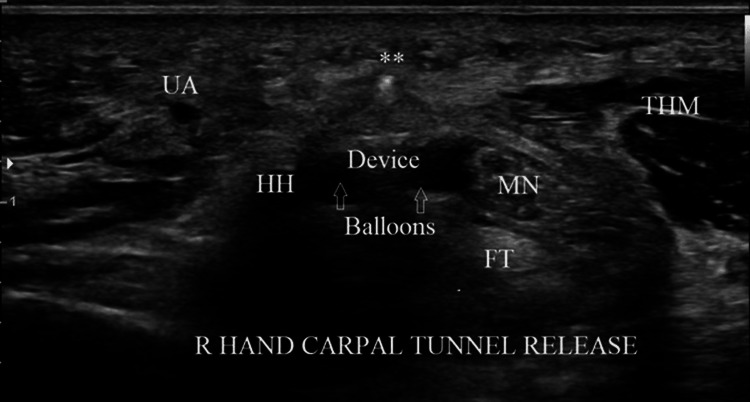
Transverse ultrasound view of the carpal tunnel, with ulnar on the left, at the level of the hook of hamate (HH). The device is positioned in the transverse safe zone (TSZ) between the median nerve (MN) radially and the hook of the hamate ulnarly. The balloons, filled with sterile saline, have been deployed to create space in the TSZ. In this view, the blade is seen in cross-section (double asterisk), appearing as a bright dot above the transverse carpal ligamen UA = ulnar artery, ThM = thenar muscles, FT = flexor tendons

Following confirmation of accurate device positioning relative to the distal TCL and surrounding neurovascular structures, the device handle was depressed to expand the balloons and create space within the TSZ. Following reconfirmation of safe and accurate device placement, the blade was deployed, and the TCL transected distal to proximal using continuous US guidance (Figures 3-4). 

The blade was then recessed, the balloons deflated, and the device removed under US visualization. The elevator was then re-inserted, and the TCL probed under direct US visualization to ensure complete transection. If incomplete transection was suspected, the device was re-inserted, and the procedure was repeated accordingly. A post-procedure scan was then completed to assess the integrity of the regional neurovascular structures. Following device removal and probing, excessive fluid was allowed to drain from the wound, and pressure was applied. The wound was covered with three to four sterile adhesive strips, a gauze pad, and a sterile transparent film dressing. A post-procedure exam was performed to assess for neurologic or tendon injuries.

Outcomes

Main outcomes of this study were complications, symptom severity and functional status scores of the Boston Carpal Tunnel Questionnaire (BCTQ-SSS, BCTQ-FSS) [24-26] and the Quick form of the Disabilities of the Arm, Shoulder, and Hand (QDASH) score [27-29]. Outcomes were reported at two weeks, one month, three months, and 12 months postprocedure.

Statistical analysis

At each postoperative time-point, BCTQ-SSS, BCTQ-FSS, and QDASH scores for the patient cohort were summarized with mean and standard error (SE). Statistical significance for pre- versus postoperative differences was analyzed using a nonparametric Wilcoxon test. The minimal clinically important differences (MCIDs) were defined as 1.14 points on the BCTQ-SSS, 0.74 points on the BCTQ-FSS, and 15 points on the QDASH [24,28]. Differences between hands performed as unilateral, simultaneous bilateral, and staged bilateral procedures were assessed using a mean difference F-test. P < .05 was considered statistically significant.

## Results

Between March 2019 and August 2020, the authors treated 200 hands in 140 patients, for which one-year follow-up was available for 123 hands (69 right, 54 left) in 88 patients (57% female). Follow-up was available for over 90% of the 123 hands at both two weeks and one month, and for 85% of hands at three months. Confirmatory electrodiagnostic testing was available for all 123 hands, of which 81 (66%) had moderately severe or severe disease. The most common comorbidities were hypertension (43%), obesity (38%), and diabetes mellitus (24%) (Table 1). The mean cross-sectional area of the median nerve was 16.5±0.5 mm^2^.

**Table 1:** Patient Characteristics Treated with Carpal Tunnel Release with Ultrasound Guidance ^a^Mean (range). ^b^Number and percentage of hands with selected comorbidities.

Comorbidity	Value
No. patients	88
No. hands	123
Female sex	50 (57%)
Age (years)^a^	66 (30 - 89)
Comorbidities^b^	
Hypertension	53 (43%)
Obesity	47 (38%)
Diabetes mellitus	30 (24%)
Inflammatory arthritis, rheumatoid arthritis, or lupus	19 (15%)
Hypothyroidism	17 (14%)
Gout	12 (10%)
Peripheral neuropathy or radiculopathy	10 (8%)
Depression or anxiety	4 (3%)
Fibromyalgia	3 (2%)
Complex regional pain syndrome	1 (1%)

Twenty-nine patients (57 hands) were treated as simultaneous bilateral procedures (one patient did not provide the 12-month symptom scores on one hand), 18 patients (25 hands) were treated with staged bilateral procedures, and 41 were treated with a unilateral procedure. All office-based procedures were successfully completed with local anesthesia. No supplementary intraoperative analgesics or anxiolytics were required. The TCL was transected using one to two blade passes in 96% of cases, with 4% requiring three blade passes. All incisions were < 5 mm in length. No intraoperative complications occurred.

**Figure 5:**
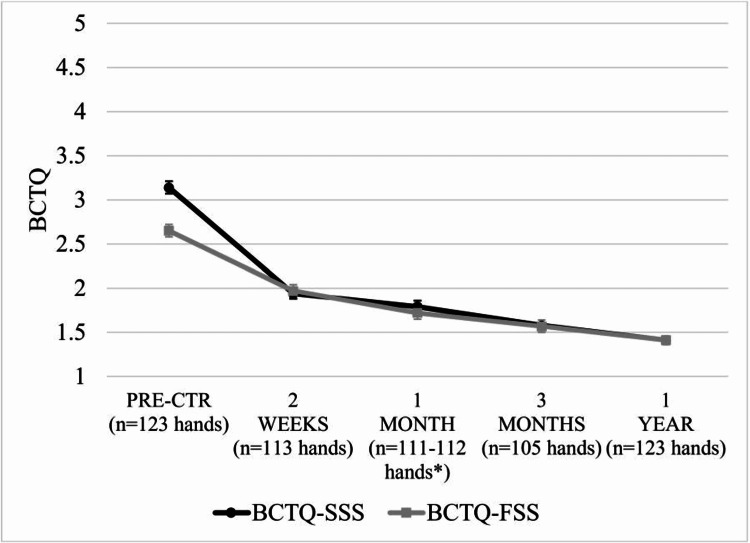
BCTQ-SSS and BCTQ-FSS scores (mean±SE) following carpal tunnel release with ultrasound guidance. Mean BCTQ-SSS and BCTQ-FSS scores were statistically significantly lower at all time points compared to pre-treatment (p < 0.001). *n=111 hands for BCTQ-SSS and n=112 hands for BCTQ-FSS. BCTQ-SSS = Boston Carpal Tunnel Symptom Severity Score, BCTQ-FSS = Boston Carpal Tunnel Functional Status Score

**Figure 6:**
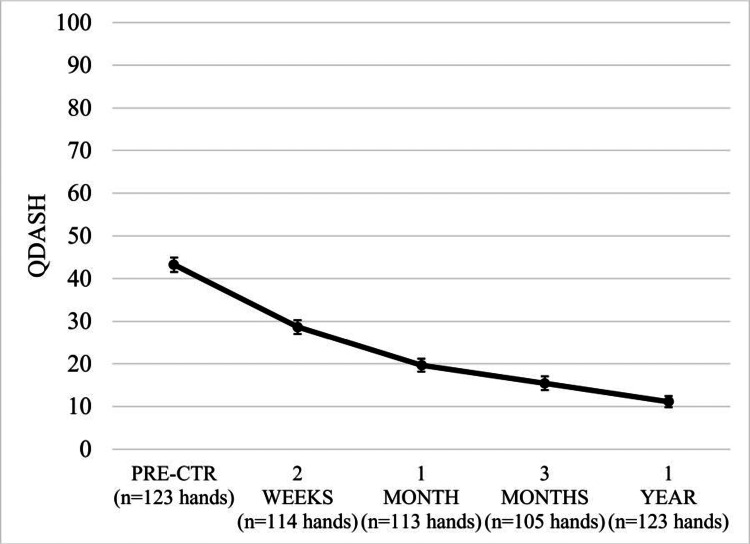
QDASH scores (mean±SE) following carpal tunnel release with ultrasound guidance. Mean QDASH scores were statistically significantly lower at all time points compared to pre-treatment (p < 0.001). QDASH = Quick form of the Disabilities of the Arm, Shoulder and Hand

BCTQ-SSS, BCTQ-FSS, and QDASH scores significantly improved by two weeks, with mean reductions of 1.19, 0.64, and 14.06, respectively (all p < 0.001) (Table 2, Figures 5-6). Mean reductions on each questionnaire exceeded the MCID by one month and scores continued to improve over the one-year follow-up period. No statistically significant differences in improvement were observed between hands treated as part of unilateral, simultaneous bilateral, or staged bilateral procedures for BCTQ or QDASH scores at any postoperative time point (p > 0.14).

**Table 2:** Patient Reported Outcomes Following Carpal Tunnel Release with Ultrasound Guidance *Statistically significant improvements in BCTQ-SSS, BCTQ-FSS and QDASH occurred at all postoperative time points. BCTQ-SSS = Boston Carpal Tunnel Symptom Severity Score, BCTQ-FSS = Boston Carpal Tunnel Functional Status Score, QDASH = Quick form of the Disabilities of the Arm, Shoulder and Hand score.

		Pre-treatment	2 Weeks	1 Month	3 Months	1 Year
n (hands, % of total hands)	BCTQ-SSS	123 (100%)	113 (92%)	111 (90%)	105 (85%)	123 (100%)
	BCTQ-FSS	123 (100%)	113 (92%)	112 (91%)	105 (85%)	123 (100%)
	QDASH	123 (100%)	114 (93%)	113 (92%)	105 (85%)	123 (100%)
Mean (Std Error)	BCTQ-SSS	3.14 (0.07)	1.94 (0.06)	1.79 (0.07)	1.58 (0.06)	1.41 (0.05)
	BCTQ-FSS	2.65 (0.07)	1.97 (0.07)	1.72 (0.07)	1.57 (0.07)	1.41 (0.05)
	QDASH	43.24 (1.67)	28.67 (1.62)	19.66 (1.51)	15.45 (1.63)	11.14 (1.28)
Mean Change from Pre-treatment (Std Error)*	BCTQ-SSS	-	-1.19 (0.07)	-1.32 (0.07)	-1.56 (0.09)	-1.73 (0.08)
	BCTQ-FSS	-	-0.64 (0.06)	-0.90 (0.07)	-1.08 (0.07)	-1.24 (0.07)
	QDASH	-	-14.06 (1.52)	-22.98 (1.56)	-27.48 (1.69)	-32.10 (1.77)

Two (2.3%) patients required a supplementary three-day supply of tramadol to control postoperative pain. One patient developed symptoms consistent with complex regional pain syndrome including unilateral arm pain, paresthesias, and trace edema two weeks postoperative. Follow-up examination showed no erythema or signs of infection. Follow-up ultrasound examination showed complete transection of the TCL, no evidence of nerve injury, and no signs of seroma or abscess formation. The patient was treated with a short course of oral prednisone and vitamin C, with symptom resolution within two weeks. The same patient subsequently had the contralateral hand released using the same office-based procedure without complication. No neurovascular injuries, tendon injuries, infections or significant post-surgical wound complications occurred in any hand. There were no recurrences or reoperations.

## Discussion

The primary finding of this study was that office-based CTR using US guidance is well-tolerated, safe, and results in statistically significant and clinically important CTS symptom improvement in a community population followed for one year postprocedure. All 123 hands were successfully treated in the office using only local anesthesia without the need for supplementary anxiolysis or analgesia. There were no intraoperative complications, and no conversions were required due to inadequate visualization. Furthermore, during the one-year follow-up period, there were no recurrences or reoperations, and only one significant complication occurred - a patient with complex regional pain syndrome unaccompanied by neurovascular injury that was promptly recognized and successfully treated.

Although traditional CTR using larger incisions has an established record of safety and effectiveness, over time there has been a trend to use smaller incisions to minimize surgical morbidity and accelerate recovery [3,6-10]. In recent years, there has been increased interest in using US to perform CTR through small incisions [9,11-22]. During CTR, US provides detailed, real-time visualization of all relevant structures in the carpal tunnel, as well as the ability to directly probe the TCL to ensure a complete transection [9,11,12,15,18-22].

To date, seven studies have documented the long-term (> one-year) safety and effectiveness of CTR using US guidance, including one prospective, randomized study demonstrating significantly faster recovery compared to mini-open CTR (mOCTR) [9,11,15,19-22]. However, none of these studies reported results from large patient cohorts (> 100 hands) treated in an office-based procedure room. Importantly, the results of the current investigation add to the existing literature by documenting that CTR using US guidance is also safe and effective at one year when performed in an office-based procedure room.

As the number of publications pertaining to CTR using US guidance continues to grow, it becomes increasingly important to address the generalizability of results with respect to typical patients encountered in clinical practice. All patients treated in the current investigation were seen within the context of a rural community practice, and the cohort with a mean age of 66 years, chronic symptoms (> six months), and a female predominance (57%) was typical of patients treated with CTR [6]. Furthermore, unlike most previously published studies, we did not exclude patients based on specific medical comorbidities (e.g., inflammatory arthritis, neuropathy). In addition, 66% of hands had moderately severe or severe disease based on electrodiagnostic criteria. These observations, in combination with performing the procedures in the office-based procedure room, support the generalizability of the current results to diverse practice settings.

Many patients with CTS present with bilateral disease and some are candidates for bilateral CTRs [30-31]. In these patients, the ability to perform simultaneous bilateral releases offers the patient the opportunity to significantly reduce the overall episode of care [18,19,30]. In the current study, 46% (57/123) of the hands were treated as part of simultaneous bilateral procedures. All patients tolerated the simultaneous procedures in the office-based procedure room, and there was no significant difference in clinical outcomes between hands treated as part of simultaneous procedures compared to unilateral or staged bilateral procedures. Only two of the previously mentioned seven long-term studies specifically report performing simultaneous bilateral releases [19,20]. Leiby et al. treated 50 hands in 25 patients in either an ambulatory surgery center or hospital operating room [19], and more recently Wang et al. treated 86 hands in 43 patients in a hospital setting [20]. As with the current study, Leiby et al. found no significant differences in outcomes between simultaneous bilateral releases and the other groups at one-year follow-up. Although Wang et al. did not specifically present outcomes based on procedure type, they did not specifically identify simultaneous bilateral procedures as being problematic [20]. Collectively, the results of these studies support the feasibility of performing simultaneous bilateral CTRs using US guidance in multiple practice settings, including the office procedure room as documented in the current study.

Study strengths include a prospective design, long-term follow-up on a large patient cohort consisting of >100 hands, excellent generalizability of results, inclusion of a large number of hands with severe disease, statistical comparison of patient outcomes with unilateral, simultaneous bilateral, and staged bilateral procedures, and use of validated patient-reported outcomes. However, several study limitations also warrant discussion. First, there was no comparison group. However, the primary purpose of this investigation was to specifically document the safety and effectiveness of office-based CTR using US guidance in our community practice. Of note, the current results are similar to those reported by Leiby et al. for 76 hands in 47 patients treated in the ambulatory surgery center or hospital operating room setting with the same US-guided CTR technique [19]. Second, the follow-up data reported in the current study were obtained primarily through mailed questionnaires and only 123 of 200 hands in 88 of 140 patients provided one-year follow-up. To the authors’ knowledge, no intraoperative or postoperative complications occurred in the remaining hands without one-year data. Third, this was a single-center study. Prospective multicenter studies of office-based CTR using US guidance are warranted to determine if comparable patient outcomes are feasible among a wider range of physician users. Finally, although validated patient-reported outcomes were utilized, no specific return to activity, return to work, or global satisfaction data were collected in this study.

## Conclusions

Office-based CTR using US guidance is safe, effective and results in significant and sustained clinical improvements at one-year follow-up within the context of a community practice. Clinically important reductions in pain and improvements in function were reported early in follow-up and maintained throughout the course of the study. When clinically indicated, simultaneous bilateral procedures were well-tolerated and resulted in similar clinical improvements as unilateral or staged bilateral procedures. Overall, office-based CTR using US guidance is an attractive minimally invasive treatment option for patients with severe or refractory CTS.
